# The Metastatic Cascade as the Basis for Liquid Biopsy Development

**DOI:** 10.3389/fonc.2020.01055

**Published:** 2020-07-21

**Authors:** Zahra Eslami-S, Luis Enrique Cortés-Hernández, Catherine Alix-Panabières

**Affiliations:** Laboratory of Rare Human Circulating Cells (LCCRH), University Medical Centre of Montpellier, UPRES EA2415, Montpellier, France

**Keywords:** metastatic cascade, liquid biopsy, circulating tumor cells, extracellular vesicles, circulating-tumor DNA, tumor educated platelets

## Abstract

The metastatic cascade describes the process whereby aggressive cancer cells leave the primary tumor, travel through the bloodstream, and eventually reach distant organs to develop one or several metastases. During the last decade, innovative technologies have exploited the recent biological knowledge to identify new circulating biomarkers for the screening and early detection of cancer, real-time monitoring of treatment response, assessment of tumor relapse risk (prognosis), identification of new therapeutic targets and resistance mechanisms, patient stratification, and therapeutic decision-making. These techniques are broadly described using the term of *Liquid Biopsy*. This field is in constant progression and is based on the detection of circulating tumor cells, circulating free nucleic acids (e.g., circulating tumor DNA), circulating tumor-derived extracellular vesicles, and tumor-educated platelets. The aim of this review is to describe the biological principles underlying the liquid biopsy concept and to discuss how functional studies can expand the clinical applications of these circulating biomarkers.

## Introduction

In 1889, Stephen Paget proposed on the basis of postmortem data that cancer cells migrate to specific organs and that this could not be explained by chance or by the blood vessel distribution. He proposed that metastatic colonization could be achieved in the presence of a compatible and reciprocal communication between tumor cells and the organ *milieu*. Accordingly, this hypothesis was named “Seed and Soil” because certain organs, such as liver, seem more “fertile” to receive cancer cells (1). However, the mechanisms behind metastasis formation are not comprehensively understood yet.

To explain the process of cancer dissemination, Fidler et al. (2) proposed a model named the “metastatic cascade” based on experiments showing that the metastatic success of a cancer cell is minimal (3). This model recapitulates the progression of cancer cells and their spreading in the body through a series of organized steps. Failure to complete any of them stops the formation of a secondary cancerous lesion (4, 5).

Briefly, the metastatic cascade initiates with the transformation and progressive growth of cells. Then, it continues with the local invasion of the surrounding tissues by primary tumor cells. This step provides a route for intravasation in already existing or new venules (after neo-angiogenesis induction) and capillaries. Subsequently, tumor cells circulate in the circulatory system (bloodstream and/or lymphatic vessels). If cancer cells manage to survive in this system, they will become trapped in the vascular walls of distant tissues where they can extravasate. Finally, if the microenvironment in these tissues offers the right conditions, cancer cells will proliferate, colonize, and form a metastatic tumor (4, 6).

Many biological factors are involved in the metastatic cascade. These factors or mechanisms are implicated in the final destination (“Soil”) or in the behavior/survival of cancer cells (“Seed”), and they vary in function of the cancer type and the patient health status (or the animals used as model), although they share some features. These factors and mechanisms are often different from the normal physiological mechanisms, and they play a vital role in cancer dissemination and survival. Historically and currently, these differences have been used to diagnose and treat cancer, such as the higher cancer cell metabolism detected by positron emission tomography, the microscopical morphological changes associated with cell transformation, and the protein expression changes associated with specific cancer types. The development of molecular techniques allows exploiting cancer-specific genetic and genomic differences for diagnostic purposes. However, these approaches have been focused and applied predominantly in primary and metastatic tumors, while the majority of the intermediate steps of the metastatic cascade have been ignored.

**Figure 1:**
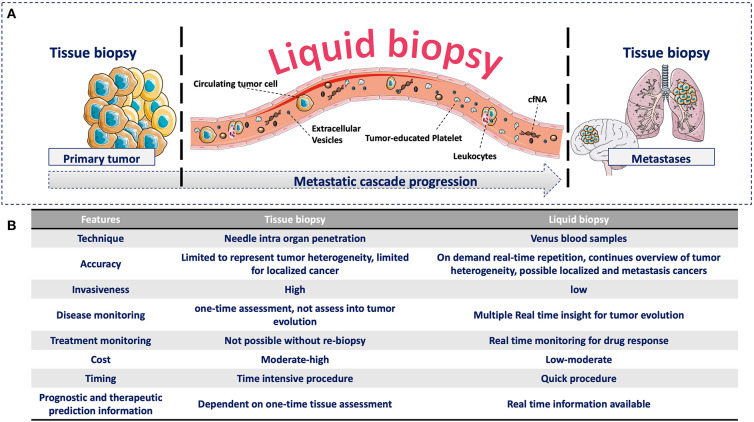
**(A)** Advantages of liquid biopsy vs. tissue biopsy during the metastatic cascade. **(A)** Compared with tissue biopsy (limitation for serial sampling and difficulty to access certain organs, such as brain and lung), liquid biopsy allows the real-time monitoring of cancer progression during the liquid phase of the metastatic cascade through the detection of different circulating analytes. These circulating analytes have specific biological functions and provide complementary information that can be continuously evaluated during cancer progression. **(B)** Comparison of tissue and liquid biopsies. Comparison of the relevant medical features of tissue biopsy and liquid biopsy.

Recently, more attention has been given to identify clinically useful cancer features using minimal invasive methods to detect analytes or biomarkers in blood. This approach has been named *liquid biopsy* (7). This is a broad term that includes the isolation, detection, and characterization of analytes released by or associated with cancer cells, such as circulating tumor cells (CTCs), circulating free nucleic acids (cfNA: circulating tumor DNA, ctDNA, and circulating free DNA, cfDNA), extracellular vesicles (EVs), and tumor-educated platelets (TEPs) (8). All of them have biological significance in the metastatic cascade and can provide clinical information that can be continuously evaluated during the natural course of the disease (Figure 1). The study of these analytes in the context of the metastatic cascade model might help to address the question formulated by Stephen Paget: “What is it that decides what organs shall suffer in a case of disseminated cancer?” (1), and more importantly, for people with cancer, “How this knowledge can help to prevent and cure cancer”?

The purpose of this review is to describe the metastatic steps with particular emphasis on the involvement of the analytes that can be tested by liquid biopsy.

## Primary Tumor Expansion and Angiogenesis

The proliferation/expansion of cancer cells is promoted by the appearance of alterations in key genes related to cell fate, cell survival, and genome integrity maintenance. These so-called driver mutations confer a selective growth advantage to the cells that harbor them and lead to the formation of an aggressive tumor (9). Recently, it has been shown that 95% of cancers have at least one driver mutation (10). During tumor cell proliferation, the passive diffusion of oxygen and nutrients reach a threshold and cannot support the tumor growth rate any longer. Consequently, some cancer cells, which are poorly adapted to survive in hypoxic conditions, undergo necrosis or apoptosis (11). However, other cancer cells develop mechanisms of protection against these harsh conditions due to tumor cell heterogeneity. Moreover, cells in the tumor microenvironment, such as cancer-associated fibroblasts, will start to secrete factors that induce angiogenesis, thus supporting the tumor continuous growth. All these factors, actively and non-actively released in the blood circulation, can be used as analytes for liquid biopsy.

For example, tumor DNA might be released in the extracellular space by necrotic tumor cells, as a consequence of the tumoral high size growth rate (which limits the diffusion of oxygen and nutrients to the central regions of the tumor). Then, DNA can reach the circulation, after neo-angiogenesis, where it is described as ctDNA. This is just a fraction of the total cfDNA in blood, but this analyte can be analyzed to identify tumor driver mutations that can be therapeutically targeted, such as mutations in epidermal growth factor receptor (EGFR) in non-small cell lung cancer (NSCLC) that are currently used in the clinic (12). These cfNA (cfDNA and ctDNA) usually include fragments with a size similar to that of DNA surrounding nucleosomes, suggesting a nuclear origin after the disruption of the nuclear and cellular membranes (13–16). However, the mechanisms by which ctDNA originates are not clear. Indeed, some studies suggested that cfDNA is actively released by the cells (17). Moreover, most cfDNA normally originates from hematopoietic precursors in the bone marrow (18, 19). Also, somatic mutant clones can appear in healthy tissue cells during normal aging (20) and could be mistaken for ctDNA. Nonetheless, the total cfDNA amount is higher in patients with cancer patients, most likely due to an increase in the ctDNA fraction (15).

The presence of physiological cfDNA with somatic mutations might hamper the use of ctDNA and cfDNA for the diagnosis of early-stage cancer (21). However, a recent study showed that ctDNA can be used for NSCLC screening because ctDNA can be differentiated from hematopoietic cfDNA by correlating the DNA fragment size (shorter fragments were associated with ctDNA). The sensitivity and specificity of this method are lower than those of low-dose CT imaging (22), the currently used screening method, and similar to what was reported for chest X-rays (23). Nonetheless, ctDNA-based screening might be exploited as marker of tumor recurrence or for detection of driver mutations, for example, by identifying first the hematopoietic somatic mutations and then discarding them in order to focus only on ctDNA (24).

On the other hand, it has been also suggested that ctDNA is actively secreted inside tumoral EVs. These vesicles can protect it from degradation in the bloodstream. Exosomes, a small EV subtype of endocytic origin, are abundant in blood samples from patients with cancer (25) and contain dsDNA (26). Moreover, it has been suggested that up to 93% of the ctDNA detected in blood is within exosomes (27). However, this was not confirmed by a recent study that used high-resolution methodologies to evaluate exosome isolation and molecular composition (17). In agreement, other studies reported that large EVs (e.g., apoptotic bodies) and not exosomes have the highest DNA cargo (28). The lack of standardized methods for EV isolation and of validated markers for their classification makes difficult to draw conclusions from most of the studies on EVs, and common guidelines have been published only recently (29). Therefore, more research is needed to address these issues.

In the context of liquid biopsy, the specific origin of cfDNA in blood is crucial because the current methods for cfDNA isolation and enrichment cannot efficiently distinguish ctDNA from other cfDNA fractions in blood. If ctDNA were associated with a specific EV type, it would be theoretically possible to isolate only the EVs containing all or most ctDNA.

The role of ctDNA or cfDNA in the metastatic cascade is unknown, and few studies have addressed this question. cfDNA might have different biological roles in function of the proteins it associated with. For example, cfDNA associated with histones can trigger a proinflammatory response related to toll-like receptors (TLR2/4), and cytotoxic effects in endothelial cells (31), whereas cfDNA released from apoptotic adipocytes induces an inflammatory reaction by increasing the accumulation of macrophages *via* TLR9 (32). However, evidence for this is still limited to *in vitro* studies, and the different methodologies used to isolate cfDNA and ctDNA make comparison among studies difficult.

More data are available on EV's role during the early steps of the metastatic cascade. During angiogenesis, exosomes facilitate the intravasation of the different liquid biopsy analytes into the bloodstream. Exosomes in the extracellular space have antiangiogenic and proangiogenic roles. For instance, *in vitro* studies have shown that miR-23a from tumor-associated exosomes secreted in hypoxic conditions has proangiogenic effects by increasing endothelial permeability and suppressing the expression of prolyl-hydroxylase 1 and 2. This leads to the accumulation of hypoxia-inducible factor 1 α (HIF-1 α) in endothelial cells (33). Accordingly, exosomes from colorectal cancer cell lines induce endothelial permeability *via* the cytoskeletal-associated RhoA/ROCK pathway (34). Moreover, in mouse models of oral cancer, microRNAs contained in EVs, such as miR-34a, miR21, and miR324, have been associated with adrenergic trans-differentiation of sensory nerves that innervate tumors upon loss of TP53 (35).

Other factors also are involved in the early steps of cancer cell dissemination, particularly several cytokines that are interesting therapeutic targets, for instance vascular endothelial growth factor (VEGF), tumor necrosis factor alpha and beta (TNF), and chemokine (C–C motif) ligand 2 (CCL2) (36). Moreover, different cancers can secrete different proteins. For example, prostate specific antigen (PSA), which is used as a blood cancer marker, is secreted by prostate cancer cells, and carcinoembryonic antigen (CEA) by colorectal cancer cells (37). These “classical” tumor markers are routinely tested in the framework of cancer management, but they often lack specificity or sensitivity. Cohen et al. (30) demonstrated that ctDNA can be combined with eight classical tumor markers in a multi-analyte liquid biopsy assay for cancer screening. This method shows promising results but needs to be validated for clinical applications (30). Therefore, these tumor markers should be considered in the liquid biopsy field (Table 1).

**Table 1:** Comparison of “Classic” tumor markers and “New” liquid biopsy analytes.

**Liquid biopsy field**			
**“Classic” tumor marker(s)**		**“New” liquid biopsy analyte(s)**	
• **CA 27.29** • **CA 19-9** • **CA15-3** • **CA 125** • **PSA** • **NSE** • **CEA** • **AFP**	• **Strength** ✓High level with progressive disease ✓Decrease rate with remission ✓Simple procedure ✓Easily obtainable specimens ✓Minimal invasive ✓Currently using in clinic • **Limitation** ✓Lack of specificity and sensitivity ✓Detectable in benign and healthy conditions ✓Do not provide information related to the metastatic cascade ✓High level only in large tumor volumes	• **CTCs** • **ctDNA** • **TEPs** • **EVs**	• **Strength** ✓Cancer specific ✓Simple procedure ✓Easily obtainable specimens ✓Minimal invasive ✓Genome, transcriptome, proteome and secretome evaluation ✓Related with metastasis cascade ✓Informative for cancer heterogeneity ✓Monitoring drug response and resistance therapy ✓High specificity and sensitivity • **Limitation** ✓Not completely validate and/or utilize in clinic ✓Lack of standardize and integrated method for detection

*Circulating analytes, such as CTCs, ctDNA, TEPs, and EVs, are already part of the liquid biopsy, and classical tumor markers also could be included. Indeed, they can be detected in serum, and the combination of classical tumor markers with circulating analytes (e.g., ctDNA) can be used to develop a multi-analyte blood test (30) as a screening method for early tumor detection*.

* AFP, Alfa fetoprotein; CA, Carbohydrate antigen; CEA, Carcinoembryonic antigen; PSA, Prostate-specific antigen; NSE, Neuron specific enolase; CTC, Circulating tumor cells; CtDNA, Circulating tumor DNA; TEPs, tumor-educated platelets; EVs, Extracellular vesicles.

On their surface, cancer cells present peptides that are absent from the normal human genome and are called neoantigens. These peptides are generated by tumor-specific non-synonymous genetic alterations and are associated with the tumor mutation burden (TMB). Moreover, they are present at the cancer cell surface in association with the major histocompatibility complex (MHC). Therefore, neoantigens can trigger an immune reaction via activation of T cell receptors (TCRs) (38). Like for the previously described analytes, it has been proposed that neoantigens, TMB, and antitumoral T cells should be monitored by liquid biopsy. For instance, the detection of neoantigens on the surface of EVs or CTCs could be used as a treatment response as predictor for immune checkpoint therapies. Similarly, the TMB could be evaluated in ctDNA (39). Gros et al. (40) showed that neoantigen-specific T cells (CD8^+^, PD-1^+^) can be identified in blood samples from patients with melanoma. This might lead to the development of personalized therapies using these T cells (40).

All the aforementioned factors might be released in the hypoxic context of rapidly growing tumors and will facilitate the next step of the metastatic cascade related to cancer cell dissemination and therefore to CTC formation. Whether or not liquid biopsy analytes have a main role in the early steps of the metastatic cascade is still unknown, but the information that they bring might be valuable for clinical applications.

## Local Invasion, Detachment, and Intravasation in Circulation

Tumor cell proliferation and neo-angiogenesis increase the chances that cancer cells reach the blood circulation. Epigenetic modifications play a major role in this step by altering the expression of genes related to cell migration, thus promoting the free movement of cells through the tissue (41). Then, some cancer cells break and cross the endothelial barrier to intravasate in the blood or lymphatic stream, thus becoming CTCs. Although this is a well-accepted idea, the exact mechanisms are not fully understood and might vary according to tumor type, anatomical location, patient health status, and cancer stage. Nevertheless, these mechanisms leave a signature in blood that can be assessed by liquid biopsy.

During local invasion, cancer cells secrete metalloproteinases that disrupt the basal membrane. Then, in order to detach from the tissue, they must acquire significant changes to survive in the harsh conditions of the bloodstream and the connective tissue (42). For instance, loss of cellular junctions in normal epithelial cells induces apoptosis, a process called “anoikis” (43). To survive this process, it has been suggested that CTCs acquire mesenchymal features through the epithelial–mesenchymal transition (EMT) process. This mechanism is physiologically used by epithelial cells during embryogenesis or wound healing to acquire a mesenchymal phenotype, migrate to reach distant regions or cross connective tissues, and then to reacquire an epithelial phenotype. This process also allows cancer cells to detach from the main tumor, modify their cytoskeleton, move within the connective tissue, and intravasate through endothelial cells (41). As one of the key features of EMT is the switch from expression of the adherent junction protein E-cadherin to N-cadherin, this might limit the use of CTCs as liquid biopsy. In fact, most methods for CTC detection are based on epithelial markers, particularly epithelial cell adhesion molecule (EpCAM). This protein is expressed on the surface of epithelial cells and is used as an enrichment surface marker by the CellSearch® System (Menarini Silicon Biosystems^©^), the only method cleared by the US Food & Drug Administration (FDA) for CTC enumeration.

Intravasation might occur at early tumor stages, and it has been suggested that early-stage dissemination is a very common phenomenon (44). However, CTCs are a rare event in most patients, even those with advanced disease (7); therefore, the chance of capturing few CTCs is minimal in these early stages. However, their detection at early stages could represent an early marker of cancer or of recurrence after treatment.

On the other hand, during late dissemination, CTC enumeration has been clinically validated as a prognostic tool in many cancer types. For example, in metastatic breast cancer, detection of > 5 CTCs per 7.5 ml of blood is associated with shorter overall survival and lower progression-free survival (45). Ongoing clinical trials assess whether CTC enumeration might guide therapeutic decision-making, particularly as a sign of treatment failure, when the number of CTCs remains high. In addition, CTCs harbor therapeutic predictive markers that in the current medical practice can only be analyzed in the tumor tissue. Some examples are PD-L1 in NSCLC (among many others) (46), HER2 in breast and stomach cancer (47), and androgen receptor variant 7 (AR-V7) in prostate cancer (48). As these markers have already shown their clinical utility when evaluated in tissue biopsy, their detection in CTCs could take the place of tissue biopsy in the future. However, not all patients have the same number of CTCs in blood. Therefore, it is crucial to determine the percentage of positive CTCs required for the correlation with the target therapy outcomes, based on the current diagnostic methods. CTC clinical implications have been extensively reviewed elsewhere (49, 50).

Nonetheless, the methods for detection, capture, and characterization of these rare cells must be improved to increase their clinical utility as liquid biopsy and possibly as a screening method in early-stage cancer.

## Embolization and Tumor Cell Survival

The next step of the metastatic cascade involves mainly CTCs and how these cells survive and adapt to the blood stream environment. This is the most critical part of the metastatic cascade, as indicated by the fact that <0.1% of the cancer cells injected in animal models are viable after 24 h (2). Moreover, clinical studies showed that liver works as a filter against viable CTCs when cancer cells transit through the portal vein (51). This step is the least characterized because CTC study in blood is very challenging and only recent methodological approaches had allowed assessing the underlying mechanisms, with clear implications for the liquid biopsy field.

Cancer cells can reach the circulation as single cells or as clusters/micro-emboli. Recent studies found that CTC clusters are formed in the tumor. Such clusters display higher metastatic potential compared with single cells, because they increase cell survival and reduce apoptosis (52). CTC clustering also induce specific changes in DNA methylation that promote stemness properties and metastasis formation (53). Moreover, CTC clustering with neutrophils promote cell-cycle progression and survival, thus favoring metastasis developments (54). Unlike single CTCs, CTC clusters might not need to go through EMT. Indeed, Gkountela et al. (53) demonstrated a specific methylation pattern that promotes expression of stemness-related genes, when CTCs are clustered, but they did not find any change in the methylation profile of EMT-related genes. Other studies also suggested that EMT might not have a role in CTC clusters (55, 56).

Although CTC clusters might display higher metastatic potential, single CTCs are present in higher number, with a clear association with prognosis and overall survival (45, 57). This suggests that single CTCs might easily escape the physiological filters such as liver and that their higher number might increase the chance of producing a metastatic tumor, despite their low effectiveness. On the other hand, CTC clusters are less likely to escape these filters but are more efficient in metastasis formation. Different cancer types might use one or both ways to disseminate, in function of their stage or location (58).

It is important to note that there is neither standardized method nor criteria to characterize CTC clusters. Moreover, the current technologies might be biased toward the detection of single CTCs (smaller) or clusters (bigger), although both CTC types might be present in the blood of patients with cancer during the disease course. Therefore, the clinical meaning of CTC clusters is not clear yet. For clinical implications, it might be necessary to fully identify the different CTC subpopulations.

CTCs can also gain physical and immune protection by interacting with platelets that can form a kind of coat around CTCs (59) shortly after their release in the blood stream. Platelets' role in cancer metastases has been already highlighted (60), whereas their potential role as circulating biomarker (i.e., TEPs) is only emerging now (61). TEPs' role as biomarker is based on the cross talk between platelets and CTCs that affects tumor cell proliferation and dissemination (62). Indeed, TEPs promote CTC survival, escape from immune surveillance, tumor–endothelium interactions, and dissemination. Reciprocally, CTCs modify the RNA profile of blood platelets and “tumor-educate” them (61). This “education” process might occur during the formation of CTC-platelet clusters, possibly through the uptake of exosomes and/or thrombin from CTCs (63). Simultaneously, platelets release different growth factors from α-granules (64) that can induce tumor cell proliferation and angiogenesis and also EMT (65). TEP clinical applications require further validation and standardization.

Micro-emboli/cluster formation protects CTCs and increases their survival in the bloodstream (52, 66, 67). This occurs by interaction of tumor CD44 with platelet P-selectin and the fibrinogen receptor GPIIb-IIIa which are involved in CTC coating by platelets (68). Platelets enhance tissue factor and P2Y12 receptor activities that contribute to EMT (69, 70). Additionally, TGFβ secretion by platelets can induce the TGFβ/SMAD and NF-κB pathways, which are the main molecular pathways implicated in EMT induction, thus increasing CTC metastatic potential (71). In turn, EMT in CTCs increases their interaction with platelets through the expression of heat shock protein 47 (HSP47), a chaperone implicated in collagen secretion and deposition that might enhance the formation of CTC clusters associated with platelets. Finally, HSP47 amplification in CTCs has been associated with a higher metastatic rate (72, 73).

## Establishment of The Tumor Microenvironment, Arrest, and Extravasation in a Target Organ

In the last step of the metastatic cascade, CTCs must interact with endothelial cells, mainly in capillaries where cancer cells get trapped. After their arrival in the endothelium, CTCs increase the permeability of the endothelial barrier, allowing their extravasation in a tissue/organ. Once in the tissue, cancer cells will grow if the local microenvironment conditions are favorable for their survival and proliferation (i.e., “fertile soil”).

Many proteins are involved in CTC arrest at the surface of endothelial cells. *In vivo* experiments in zebrafish cancer models have shown that CTC arrest occurs in two steps: first by weak interactions *via* CD44 and the integrin αVβ3 and then by stronger attachment *via* the integrin α5β1 (74). Hydrodynamic forces also influence CTC arrest. For instance, the brain supratentorial regions are more susceptible to metastases due to the local low perfusion and low flow pressure (75). Nevertheless, the involved mechanisms might vary according to the cancer type and the target organ. Moreover, CTC clusters might get trapped in arterioles or venules due to their larger size, and this might play a bigger role than protein interactions in their arrest at the surface of endothelial cells.

Then, blocked CTCs must extravasate to the tissue. As observed during intravasation, CTCs might release EVs that modify the cytoskeleton of endothelial cells and increase their permeability, thus allowing the crossing of the endothelial barrier (34, 76). Platelets also might be involved in this process. Indeed, CD97-expressing CTCs activate platelets to release their granules that might first increase coagulation around the cells and then promote endothelial permeabilization through secretion of ATP that mediates endothelial cell junction dynamics (77).

Finally, CTCs must arrive in a favorable niche, known as pre-metastatic niche. Exosomes are crucial for the induction of the pre-metastatic niche. Hoshino et al. (78) showed that tumor-associated exosomes present cancer-specific integrin profiles that are associated with formation of the pre-metastatic niche in specific organs, for instance integrin αvβ5 and liver, or α6β4, and α6β1 and lung (i.e., cancer cell organotropism) (79). Uptake by target cells in these organs stimulates the expression of genes that promote migration and inflammation in Küpfer cells and lung fibroblasts, or cell migration-inducing and hyaluronan-binding protein (CEMIP) in the brain (80), thus finalizing the metastatic cascade. However, a favorable pre-metastatic niche is not enough to maintain cancer cell proliferation. Therefore, CTCs must have additional features to sustain their growth, like stem cells (81). For instance, the expression of aldehyde dehydrogenase (a tumor-initiating stem cell marker) together with other surface markers can suggest which CTCs is competent for brain metastasis (82). Detection of these exosome- and CTC-related markers by liquid biopsy might allow predicting specific metastatic sites or even developing target therapies.

## Conclusion

**Figure 2:**
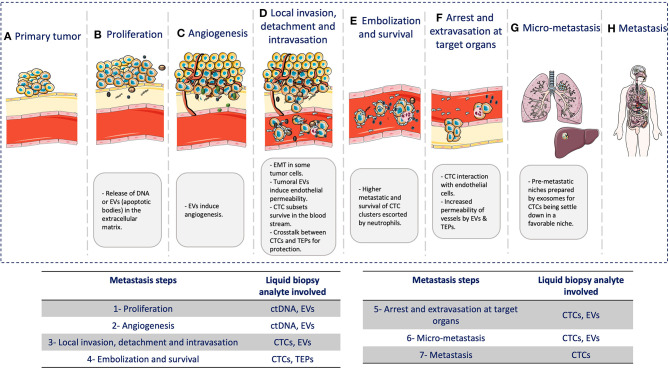
Liquid biopsy and the metastatic cascade. During the metastatic cascade, different analytes are involved: **(A)** Primary tumor: tumor growth at the primary site. **(B)** Cancer cell proliferation: release of DNA from necrotic/apoptotic cells when due to the continuous cancer cell proliferation, passive diffusion of oxygen and nutrients is no longer enough to cover the cell requirement. **(C)** Angiogenesis: EVs are involved in neo-angiogenesis. **(D)** Local invasion, detachment, and intravasation: Cancer cells acquire significant changes to survive the physical interactions and mechanical forces in blood and tissues. Tumor cells also undergo epithelial-to-mesenchymal transition (EMT) to move within the connective tissue and then secrete exosomes that contribute to induce endothelial permeability for intravasation through endothelial cells. **(E)** Embolization and survival: CTCs can survive and adapt in the blood by forming CTC clusters/micro-emboli that display higher metastatic potential by increasing cancer cell survival and reducing apoptosis. Clustering with neutrophils can also promote cell-cycle progression and survival in CTCs, further promoting metastatic development. CTCs also gain physical and immune protection by interacting with platelets. **(F)** Arrest and extravasation in the target organ: CTCs interact with endothelial cells, mainly in capillaries where they become trapped. CTCs and platelets might release EVs that can modify the cytoskeleton of endothelial cells and increase the blood vessel permeability, thus allowing CTC extravasation. **(G)** Micro-metastases: CTCs need a favorable niche. Before CTC arrival, this niche is called pre-metastatic niche, and exosomes play a major role in its preparation. **(H)** Metastases: CTCs that arrive in distant organs, such as liver or lung, will form a metastatic lesion, thus concluding the metastatic cascade.

In this review, we highlighted the role of different liquid biopsy analytes to understand the biology of the metastatic cascade (Figure 2). Although their role is fundamental in most of the steps of this process, other factors are involved as well. However, clinical decision-making based on the metastatic cascade biology must be the final goal of liquid biopsy. The increasing knowledge of the whole metastatic process (cellular and molecular) will allow the identification of new biomarkers and analytes that can be detected during the entire disease course, by taking advantage of the fact the cancer cells disseminate mainly through the blood. Therefore, it is humanly and technically possible to monitor in real time cancer progression in a patient.

There are many more factors than those described in this review that can be used as liquid biopsy. For instance, leukocytes might provide crucial information on how the immune system is reacting against the cancer. This is becoming very important due to the development of immune therapy. This is just an example of how the metastatic cascade model can help to predict new biomarkers in the liquid biopsy field and to offer possible alternative solutions in case of technical limitations. Additionally, the identification of new biomarkers will promote the development of targeted therapies against the metastatic process and not just against the primary tumor.

**Figure 3:**
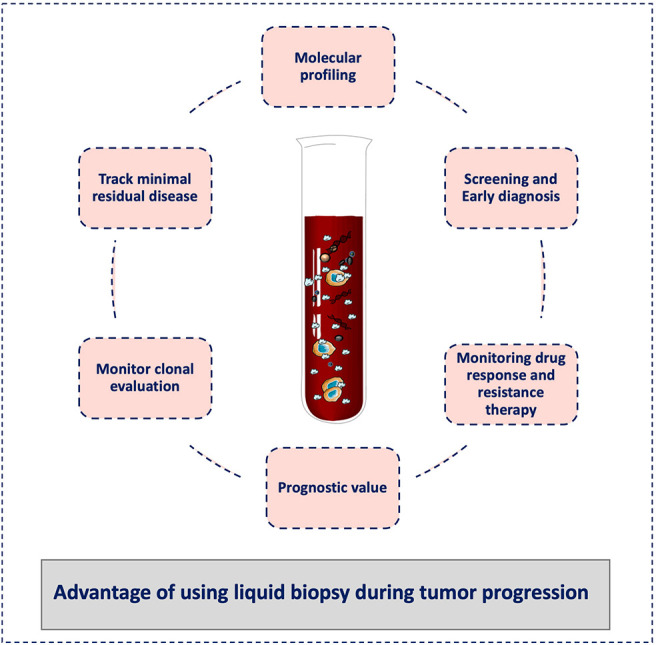
Advantages of liquid biopsy to monitor tumor progression. Liquid biopsy has broad potential applications for cancer diagnosis and treatment, including screening for early diagnosis, study of tumor heterogeneity, and clonal evolution, and detection of minimal residual disease. During cancer progression, liquid biopsy might help clinicians to stratify patients for treatment personalization and to monitor the treatment response and the development of resistance. It can also be used for the tumor molecular characterization, and its minimally invasive nature allows repeat sampling to monitor changes over time without the need for a tissue biopsy.

Finally, a liquid biopsy test should not be seen as a method to detect just one specific biomarker or analyte but as a comprehensive approach for the selection and combination of different biological clues during cancer progression. Similar to tissue biopsy, these tests should not be understood only as “positive or negative” tests, but they should be chosen and analyzed in the clinical context of each single patient in order to provide a real-time personalized medicine approach. In this way, liquid biopsy can become the tool to monitor the entire metastatic cascade avoiding the limitations of tissue biopsy sampling of single tumors, which might not be representative of the whole evolution of the cancer disease (Figure 3).

## Author Contributions

All authors contributed in the design and wrote of this manuscript.

## Conflict of Interest

CA-P is one of the patent holders (US Patent Number 16,093,934) for detecting and/or characterizing circulating tumor cells. The remaining authors declare that the research was conducted in the absence of any commercial or financial relationships that could be construed as a potential conflict of interest.

## References

[1] Paget S. The distribution of secondary growths in cancer of the breast. Lancet. (1889) 133:571–3. 10.1016/S0140-6736(00)49915-0 2673568

[2] Fidler IJ. Metastasis: quantitative analysis of distribution and fate of tumor emboli labeled with 125 I-5-iodo-2'-deoxyuridine. J Natl Cancer Inst. (1970) 45:773–82. 5513503

[3] Chambers AF Naumov GN Vantyghem SA Tuck AB. Molecular biology of breast cancer metastasis. Clinical implications of experimental studies on metastatic inefficiency. Breast Cancer Res. (2000) 2:400–7. 10.1186/bcr86 11250733 PMC138662

[4] Fidler IJ. The pathogenesis of cancer metastasis: the “seed and soil” hypothesis revisited. Nat Rev Cancer. (2003) 3:453–8. 10.1038/nrc1098 12778135

[5] Poste G Fidler IJ. The pathogenesis of cancer metastasis. Nature. (1980) 283:139–46. 10.1038/283139a0 6985715

[6] Lambert AW Pattabiraman DR Weinberg RA. Emerging biological principles of metastasis. Cell. (2017) 168:670–91. 10.1016/j.cell.2016.11.037 28187288 PMC5308465

[7] Pantel K Alix-Panabières C. Liquid biopsy and minimal residual disease — latest advances and implications for cure. Nat Rev Clin Oncol. (2019) 16:409–24. 10.1038/s41571-019-0187-3 30796368

[8] Eslami-S Z Cortes-Hernandez LE Cayrefourcq L Alix-Panabieres C. The different facets of liquid biopsy: a Kaleidoscopic view. Cold Spring Harb Perspect Med. (2019) 10:a037333. 10.1101/cshperspect.a037333 31548226 PMC7263091

[9] Vogelstein B Papadopoulos N Velculescu VE Zhou S Diaz LAJ Kinzler KW. Cancer genome landscapes. Science. (2013) 339:1546–58. 10.1126/science.1235122 23539594 PMC3749880

[10] Campbell P Getz G Korbel J Joshua M Jennings J Stein L Pan-cancer analysis of whole genomes. Nature. (2020) 578:82–93. 10.1038/s41586-020-1969-6 32025007 PMC7025898

[11] Al Tameemi W Dale TP Al-Jumaily RMK Forsyth NR. Hypoxia-modified cancer cell metabolism. Front cell Dev Biol. (2019) 7:4. 10.3389/fcell.2019.00004 30761299 PMC6362613

[12] Merker JD Oxnard GR Compton C Diehn M Hurley P Lazar AJ Circulating tumor DNA analysis in patients with cancer: American society of clinical oncology and college of American pathologists joint review. J Clin Oncol. (2018) 36:1631–41. 10.1200/JCO.2017.76.8671 29504847

[13] Lo YMD Chan KCA Sun H Chen EZ Jiang P Lun FMF. Maternal plasma DNA sequencing reveals the genome-wide genetic and mutational profile of the fetus. Sci Transl Med. (2010) 2:61ra91 LP. 10.1126/scitranslmed.3001720 21148127

[14] Thierry AR Mouliere F Gongora C Ollier J Robert B Ychou M. Origin and quantification of circulating DNA in mice with human colorectal cancer xenografts. Nucleic Acids Res. (2010) 38:6159–75. 10.1093/nar/gkq421 20494973 PMC2952865

[15] Kustanovich A Schwartz R Peretz T Grinshpun A. Life and death of circulating cell-free DNA. Cancer Biol Ther. (2019) 20:1057–67. 10.1080/15384047.2019.1598759 30990132 PMC6606043

[16] Lampignano R Neumann MHD Weber S Kloten V Herdean A Voss T. Multicenter evaluation of circulating cell-free DNA extraction and downstream analyses for the development of standardized (pre)analytical work flows. Clin Chem. (2019) 66:clinchem.2019.306837. 10.1373/clinchem.2019.306837 31628139

[17] Jeppesen DK Fenix AM Franklin JL Higginbotham JN Zhang Q Zimmerman LJ. Reassessment of exosome composition. Cell. (2019) 177:428–45.e18. 10.1016/j.cell.2019.02.029 30951670 PMC6664447

[18] Sun K Jiang P Chan KCA Wong J Cheng YKY Liang RHS. Plasma DNA tissue mapping by genome-wide methylation sequencing for noninvasive prenatal, cancer, and transplantation assessments. Proc Natl Acad Sci USA. (2015) 112:E5503–12. 10.1073/pnas.1508736112 26392541 PMC4603482

[19] Razavi P Li BT Brown DN Jung B Hubbell E Shen R. High-intensity sequencing reveals the sources of plasma circulating cell-free DNA variants. Nat Med. (2019) 25:1928–37. 10.1038/s41591-019-0652-7 31768066 PMC7061455

[20] Martincorena I Fowler JC Wabik A Lawson ARJ Abascal F Hall MWJ. Somatic mutant clones colonize the human esophagus with age. Science. (2018) 362:911–7. 10.1126/science.aau3879 30337457 PMC6298579

[21] Mattox AK Bettegowda C Zhou S Papadopoulos N Kinzler KW Vogelstein B. Applications of liquid biopsies for cancer. Sci Transl Med. (2019) 11:eaay1984. 10.1126/scitranslmed.aay1984 31462507

[22] Chabon JJ Hamilton EG Kurtz DM Esfahani MS Moding EJ Stehr H. Integrating genomic features for non-invasive early lung cancer detection. Nature. (2020) 580:245–51. 10.1038/s41586-020-2140-0 32269342 PMC8230734

[23] Gavelli G Giampalma E. Sensitivity and specificity of chest X-ray screening for lung cancer: review article. Cancer. (2000) 89(Suppl. 11):2453–6. 10.1002/1097-01422000120189:112453::aid-cncr213.3.co;2-d 11147625

[24] Leal A van Grieken NCT Palsgrove DN Phallen J Medina JE Hruban C. White blood cell and cell-free DNA analyses for detection of residual disease in gastric cancer. Nat Commun. (2020) 11:525. 10.1038/s41467-020-14310-3 31988276 PMC6985115

[25] Kalluri R LeBleu VS. The biology, function, and biomedical applications of exosomes. Science. (2020) 367:eaau6977. 10.1126/science.aau6977 32029601 PMC7717626

[26] Thakur BK Zhang H Becker A Matei I Huang Y Costa-Silva B. Double-stranded DNA in exosomes: a novel biomarker in cancer detection. Cell Res. (2014) 24:766–9. 10.1038/cr.2014.44 24710597 PMC4042169

[27] Fernando MR Jiang C Krzyzanowski GD Ryan WL. New evidence that a large proportion of human blood plasma cell-free DNA is localized in exosomes. PLoS ONE. (2017) 12:e0183915. 10.1371/journal.pone.0183915 28850588 PMC5574584

[28] Vagner T Spinelli C Minciacchi VR Balaj L Zandian M Conley A. Large extracellular vesicles carry most of the tumour DNA circulating in prostate cancer patient plasma. J Extracell Vesicles. (2018) 7:1505403. 10.1080/20013078.2018.1505403 30108686 PMC6084494

[29] Thery C Witwer KW Aikawa E Alcaraz MJ Anderson JD Andriantsitohaina R. Minimal information for studies of extracellular vesicles 2018 (MISEV2018): a position statement of the International society for extracellular vesicles and update of the MISEV2014 guidelines. J Extracell Vesicles. (2018) 7:1535750. 10.1080/20013078.2018.1461450 30637094 PMC6322352

[30] Cohen JD Li L Wang Y Thoburn C Afsari B Danilova L. Detection and localization of surgically resectable cancers with a multi-analyte blood test. Science. (2018) 359:926–30. 10.1126/science.aar3247 29348365 PMC6080308

[31] Xu J Zhang X Pelayo R Monestier M Ammollo CT Semeraro F. Extracellular histones are major mediators of death in sepsis. Nat Med. (2009) 15:1318–21. 10.1038/nm.2053 19855397 PMC2783754

[32] Nishimoto S Fukuda D Higashikuni Y Tanaka K Hirata Y Murata C. Obesity-induced DNA released from adipocytes stimulates chronic adipose tissue inflammation and insulin resistance. Sci Adv. (2016) 2:e1501332. 10.1126/sciadv.1501332 27051864 PMC4820373

[33] Hsu YL Hung JY Chang WA Lin YS Pan YC Tsai PH. Hypoxic lung cancer-secreted exosomal miR-23a increased angiogenesis and vascular permeability by targeting prolyl hydroxylase and tight junction protein ZO-1. Oncogene. (2017) 36:4929–42. 10.1038/onc.2017.105 28436951

[34] Schillaci O Fontana S Monteleone F Taverna S Di Bella MA Di Vizio D. Exosomes from metastatic cancer cells transfer amoeboid phenotype to non-metastatic cells and increase endothelial permeability: their emerging role in tumor heterogeneity. Sci Rep. (2017) 7:4711. 10.1038/s41598-017-05002-y 28680152 PMC5498501

[35] Amit M Takahashi H Dragomir MP Lindemann A Gleber-Netto FO Pickering CR. Loss of p53 drives neuron reprogramming in head and neck cancer. Nature. (2020) 578:449–54. 10.1038/s41586-020-1996-3 32051587 PMC9723538

[36] Maru Y. Premetastasis. Cold Spring Harb Perspect Med. (2019) a036897. [Epub ahead of print]. 10.1101/cshperspect.a036897. 31615874 PMC7605236

[37] Holdenrieder S Pagliaro L Morgenstern D Dayyani F. Clinically meaningful use of blood tumor markers in oncology. Biomed Res Int. (2016) 2016:9795269. 10.1155/2016/9795269 28042579 PMC5155072

[38] Jiang T Shi T Zhang H Hu J Song Y Wei J. Tumor neoantigens: from basic research to clinical applications. J Hematol Oncol. (2019) 12:93. 10.1186/s13045-019-0787-5 31492199 PMC6731555

[39] Hofman P Heeke S Alix-Panabières C Pantel K. Liquid biopsy in the era of immuno-oncology: is it ready for prime-time use for cancer patients? Ann Oncol. (2019) 30:1448–59. 10.1093/annonc/mdz196 31228184

[40] Gros A Parkhurst MR Tran E Pasetto A Robbins PF Ilyas S. Prospective identification of neoantigen-specific lymphocytes in the peripheral blood of melanoma patients. Nat Med. (2016) 22:433–8. 10.1038/nm.4051 26901407 PMC7446107

[41] Dongre A Weinberg RA. New insights into the mechanisms of epithelial-mesenchymal transition and implications for cancer. Nat Rev Mol Cell Biol. (2019) 20:69–84. 10.1038/s41580-018-0080-4 30459476

[42] Wirtz D Konstantopoulos K Searson PC. The physics of cancer: the role of physical interactions and mechanical forces in metastasis. Nat Rev Cancer. (2011) 11:512–22. 10.1038/nrc3080 21701513 PMC3262453

[43] Gilmore AP. Anoikis. Cell Death Differ. (2005) 12:1473–7. 10.1038/sj.cdd.4401723 16247493

[44] Hu Z Ding J Ma Z Sun R Seoane JA Scott Shaffer J. Quantitative evidence for early metastatic seeding in colorectal cancer. Nat Genet. (2019) 51:1113–22. 10.1038/s41588-019-0423-x 31209394 PMC6982526

[45] Bidard F-C Peeters DJ Fehm T Nolé F Gisbert-Criado R Mavroudis D. Clinical validity of circulating tumour cells in patients with metastatic breast cancer: a pooled analysis of individual patient data. Lancet Oncol. (2014) 15:406–14. 10.1016/S1470-2045(14)70069-5 24636208

[46] Mazel M Jacot W Pantel K Bartkowiak K Topart D Cayrefourcq L. Frequent expression of PD-L1 on circulating breast cancer cells. Mol Oncol. (2015) 9:1773–82. 10.1016/j.molonc.2015.05.009 26093818 PMC5528721

[47] Riethdorf S Müller V Zhang L Rau T Loibl S Komor M. Detection and HER2 expression of circulating tumor cells: prospective monitoring in breast cancer patients treated in the neoadjuvant GeparQuattro trial. Clin Cancer Res. (2010) 16:2634–45. 10.1158/1078-0432.CCR-09-2042 20406831

[48] Scher HI Lu D Schreiber NA Louw J Graf RP Vargas HA. Association of AR-V7 on circulating tumor cells as a treatment-specific biomarker with outcomes and survival in castration-resistant prostate Cancer. JAMA Oncol. (2016) 2:1441–9. 10.1001/jamaoncol.2016.1828 27262168 PMC5206761

[49] Eslami-S Z Cortés-Hernández LE Alix-Panabières C Circulating tumor cells: moving forward into clinical applications. Precis Cancer Med March. (2020) 3:4 10.21037/pcm.2019.11.07

[50] Cortés-Hernández LE Eslami-SZ Pantel K Alix-Panabières C. Molecular and functional characterization of circulating tumor cells: from discovery to clinical application. Clin Chem. (2019) 66:303586. 10.1373/clinchem.2019.303586 31811001

[51] Denève E Riethdorf S Ramos J Nocca D Coffy A Daurès JP. Capture of viable circulating tumor cells in the liver of colorectal cancer patients. Clin Chem. (2013) 59:1384–92. 10.1373/clinchem.2013.202846 23695297

[52] Aceto N Bardia A Miyamoto DT Donaldson MC Wittner BS Spencer JA. Circulating tumor cell clusters are oligoclonal precursors of breast cancer metastasis. Cell. (2014) 158:1110–22. 10.1016/j.cell.2014.07.013 25171411 PMC4149753

[53] Gkountela S Castro-Giner F Szczerba BM Vetter M Landin J Scherrer R. Circulating tumor cell clustering shapes DNA methylation to enable metastasis seeding. Cell. (2019) 176:98–112.e14. 10.1016/j.cell.2018.11.046 30633912 PMC6363966

[54] Szczerba BM Castro-Giner F Vetter M Krol I Gkountela S Landin J. Neutrophils escort circulating tumour cells to enable cell cycle progression. Nature. (2019) 566:553–7. 10.1038/s41586-019-0915-y 30728496

[55] Hong SM Jung D Kiemen A Gaida MM Yoshizawa T Braxton AM Three-dimensional visualization of cleared human pancreas cancer reveals that sustained epithelial-to-mesenchymal transition is not required for venous invasion. Mod Pathol. (2019) 33:639–47. 10.1038/s41379-019-0409-3 31700162 PMC10548439

[56] Padmanaban V Krol I Suhail Y Szczerba BM Aceto N Bader JS. E-cadherin is required for metastasis in multiple models of breast cancer. Nature. (2019) 573:439–44. 10.1038/s41586-019-1526-3 31485072 PMC7365572

[57] Cabel L Proudhon C Gortais H Loirat D Coussy F Pierga JY. Circulating tumor cells: clinical validity and utility. Int J Clin Oncol. (2017) 22:421–30. 10.1007/s10147-017-1105-2 28238187

[58] Tissot T Massol F Ujvari B Alix-Panabieres C Loeuille N Thomas F. Metastasis and the evolution of dispersal. Proc Biol Sci. (2019) 286:20192186. 10.1098/rspb.2019.2186 31771479 PMC6939260

[59] Jiang X Wong KHK Khankhel AH Zeinali M Reategui E Phillips MJ. Microfluidic isolation of platelet-covered circulating tumor cells. Lab Chip. (2017) 17:3498–503. 10.1039/C7LC00654C 28932842 PMC5690580

[60] Gasic GJ Gasic TB Galanti N Johnson T Murphy S. Platelet-tumor-cell interactions in mice. The role of platelets in the spread of malignant disease. Int J cancer. (1973) 11:704–18. 10.1002/ijc.2910110322 4801854

[61] Best MG Sol N Kooi I Tannous J Westerman BA Rustenburg F. RNA-Seq of tumor-educated platelets enables blood-based pan-cancer, multiclass, and molecular pathway cancer diagnostics. Cancer Cell. (2015) 28:666–76. 10.1016/j.ccell.2015.09.018 26525104 PMC4644263

[62] Kuznetsov HS Marsh T Markens BA Castano Z Greene-Colozzi A Hay SA. Identification of luminal breast cancers that establish a tumor-supportive macroenvironment defined by proangiogenic platelets and bone marrow-derived cells. Cancer Discov. (2012) 2:1150–65. 10.1158/2159-8290.CD-12-0216 22896036 PMC3517696

[63] Heeke S Mograbi B Alix-Panabières C Hofman P. Never travel alone: the crosstalk of circulating tumor cells and the blood microenvironment. Cells. (2019) 8:714. 10.3390/cells8070714 31337010 PMC6678604

[64] Menter DG Tucker SC Kopetz S Sood AK Crissman JD Honn K V Platelets and cancer: a casual or causal relationship: revisited. Cancer Metastasis Rev. (2014) 33:231–69. 10.1007/s10555-014-9498-0 24696047 PMC4186918

[65] McAllister SS Weinberg RA. The tumour-induced systemic environment as a critical regulator of cancer progression and metastasis. Nat Cell Biol. (2014) 16:717–27. 10.1038/ncb3015 25082194 PMC6220424

[66] Hou JM Krebs MG Lancashire L Sloane R Backen A Swain RK. Clinical significance and molecular characteristics of circulating tumor cells and circulating tumor microemboli in patients with small-cell lung cancer. J Clin Oncol. (2012) 30:525–32. 10.1200/JCO.2010.33.3716 22253462

[67] Giuliano M Shaikh A Lo HC Arpino G De Placido S Zhang XH. Perspective on circulating tumor cell clusters: why it takes a village to metastasize. Cancer Res. (2018) 78:845–52. 10.1158/0008-5472.CAN-17-2748 29437766

[68] Gay LJ Felding-Habermann B. Contribution of platelets to tumour metastasis. Nat Rev Cancer. (2011) 11:123–34. 10.1038/nrc3004 21258396 PMC6894505

[69] Wang Y Sun Y Li D Zhang L Wang K Zuo Y. Platelet P2Y12 is involved in murine pulmonary metastasis. PLoS ONE. (2013) 8:e80780. 10.1371/journal.pone.0080780 24236201 PMC3827483

[70] Orellana R Kato S Erices R Bravo ML Gonzalez P Oliva B. Platelets enhance tissue factor protein and metastasis initiating cell markers, and act as chemoattractants increasing the migration of ovarian cancer cells. BMC Cancer. (2015) 15:290. 10.1186/s12885-015-1304-z 25886038 PMC4410584

[71] Labelle M Begum S Hynes RO. Direct signaling between platelets and cancer cells induces an epithelial-mesenchymal-like transition and promotes metastasis. Cancer Cell. (2011) 20:576–90. 10.1016/j.ccr.2011.09.009 22094253 PMC3487108

[72] Xiong G Chen J Zhang G Wang S Kawasaki K Zhu J. Hsp47 promotes cancer metastasis by enhancing collagen-dependent cancer cell-platelet interaction. Proc Natl Acad Sci USA. (2020) 117:3748–58. 10.1073/pnas.1911951117 32015106 PMC7035603

[73] Zhu J Xiong G Fu H Evers BM Zhou BP Xu R. Chaperone Hsp47 drives malignant growth and invasion by modulating an ECM gene network. Cancer Res. (2015) 75:1580–91. 10.1158/0008-5472.CAN-14-1027 25744716 PMC4401637

[74] Osmani N Follain G García León MJ Lefebvre O Busnelli I Larnicol A. Metastatic tumor cells exploit their adhesion repertoire to counteract shear forces during intravascular arrest. Cell Rep. (2019) 28:2491–500.e5. 10.1016/j.celrep.2019.07.102 31484062

[75] Follain G Osmani N Azevedo AS Allio G Mercier L Karreman MA. Hemodynamic forces tune the arrest, adhesion, and extravasation of circulating tumor cells. Dev Cell. (2018) 45:33–52.e12. 10.1016/j.devcel.2018.02.015 29634935

[76] Peinado H Alečković M Lavotshkin S Matei I Costa-Silva B Moreno-Bueno G Melanoma exosomes educate bone marrow progenitor cells toward a pro-metastatic phenotype through MET. Nat Med. (2012) 18:883–91. 10.1038/nm.2753 22635005 PMC3645291

[77] Ward Y Lake R Faraji F Sperger J Martin P Gilliard C. Platelets promote metastasis via binding tumor CD97 leading to bidirectional signaling that coordinates transendothelial migration. Cell Rep. (2018) 23:808–22. 10.1016/j.celrep.2018.03.092 29669286 PMC6574118

[78] Hoshino A Costa-Silva B Shen TL Rodrigues G Hashimoto A Tesic Mark M. Tumour exosome integrins determine organotropic metastasis. Nature. (2015) 527:329–35. 10.1038/nature15756 26524530 PMC4788391

[79] Costa-Silva B Aiello NM Ocean AJ Singh S Zhang H Thakur BK. Pancreatic cancer exosomes initiate pre-metastatic niche formation in the liver. Nat Cell Biol. (2015) 17:816–26. 10.1038/ncb3169 25985394 PMC5769922

[80] Rodrigues G Hoshino A Kenific CM Matei IR Steiner L Freitas D. Tumour exosomal CEMIP protein promotes cancer cell colonization in brain metastasis. Nat Cell Biol. (2019) 21:1403–12. 10.1038/s41556-019-0404-4 31685984 PMC7354005

[81] Agnoletto C Corrà F Minotti L Baldassari F Crudele F Cook JW. Heterogeneity in circulating tumor cells: the relevance of the stem-cell subset. Cancers. (2019) 11:483. 10.3390/cancers11040483 30959764 PMC6521045

[82] Zhang L Ridgway LD Wetzel MD Ngo J Yin W Kumar D. The identification and characterization of breast cancer CTCs competent for brain metastasis. Sci Transl Med. (2013) 5:180ra48. 10.1126/scitranslmed.3005109 23576814 PMC3863909

